# DNA Sequencing Reveals the Midgut Microbiota of Diamondback Moth, *Plutella xylostella* (L.) and a Possible Relationship with Insecticide Resistance

**DOI:** 10.1371/journal.pone.0068852

**Published:** 2013-07-19

**Authors:** Xiaofeng Xia, Dandan Zheng, Huanzi Zhong, Bingcai Qin, Geoff M. Gurr, Liette Vasseur, Hailan Lin, Jianlin Bai, Weiyi He, Minsheng You

**Affiliations:** 1 Institute of Applied Ecology, Fujian Agriculture and Forestry University, Fuzhou, China; 2 Key Laboratory of Integrated Pest Management for Fujian-Taiwan Crops, Ministry of Agriculture, Fuzhou, China; 3 Centers for Disease Control and Prevention, Fuzhou, China; 4 BGI-Shenzhen, Shenzhen, China; 5 EH Graham Centre, Charles Sturt University, Orange, New South Wales, Australia; 6 Department of Biological Sciences, Brock University, St. Catharines, Ontario, Canada; Volcani Center, Israel

## Abstract

**Background:**

Insect midgut microbiota is important in host nutrition, development and immune response. Recent studies indicate possible links between insect gut microbiota and resistance to biological and chemical toxins. Studies of this phenomenon and symbionts in general have been hampered by difficulties in culture-based approach. In the present study, DNA sequencing was used to examine the midgut microbiota of diamondback moth (DBM), *Plutella xylostella* (L.), a destructive pest that attacks cruciferous crops worldwide. Its ability to develop resistance to many types of synthetic insecticide and even *Bacillus thuringiensis* toxins makes it an important species to study.

**Methodology/Principal Findings:**

Bacteria of the DBM larval midgut in a susceptible and two insecticide (chlorpyrifos and fipronil) resistant lines were examined by Illumina sequencing sampled from an insect generation that was not exposed to insecticide. This revealed that more than 97% of the bacteria were from three orders: Enterobacteriales, Vibrionales and Lactobacillales. Both insecticide-resistant lines had more Lactobacillales and the much scarcer taxa Pseudomonadales and Xanthomonadales with fewer Enterobacteriales compared with the susceptible strain. Consistent with this, a second study observed an increase in the proportion of Lactobacillales in the midgut of DBM individuals from a generation treated with insecticides.

**Conclusions/Significance:**

This is the first report of high-throughput DNA sequencing of the entire microbiota of DBM. It reveals differences related to inter- and intra-generational exposure to insecticides. Differences in the midgut microbiota among susceptible and insecticide-resistant lines are independent of insecticide exposure in the sampled generations. While this is consistent with the hypothesis that Lactobacillales or other scarcer taxa play a role in conferring DBM insecticide resistance, further studies are necessary to rule out other possibilities. Findings constitute the basis for future molecular work on the functions of insect midgut microbiota taxa and their possible role in conferring host resistance to toxins.

## Introduction

The diamondback moth (DBM), *Plutella xylostella*(L.) (Lepidoptera: Yponomeutidae), is one of the most destructive pests of cruciferous crops, particularly attacking economically important vegetables, such as cabbage, broccoli, and cauliflower [1]. A recent estimate of the total costs associated with damage and management of DBM worldwide was 4–5 billion USD per annum [2]. This lepidopteran is difficult to control because it has developed resistance to all of the insecticides used against it as well as to toxins of the biological control agent *Bacillus thuringiensis* (Bt), which are also expressed by crops genetically engineered for insect resistance [1], [3].

Insecticide resistance is a serious worldwide concern and research is needed to understand the various mechanisms used by pests to develop such resistance. Several studies have investigated how DBM can become resistant to Bt and other insecticides. In their review article, Pardo-Lopoez *et al.* noted different types of mutations that have been found in DBM leading to its resistance to Bt toxins [4]. More generally, insects are known to employ detoxification enzymes [5] and systems for toxin excretion [6]. Recent work has also indicated insect symbiont-mediated insecticide resistance. The symbiotic microorganisms of insects have long been known to have significant roles in host mating preference [7], resistance to parasitism [8], plant specialization [9], [10], longevity [11], and protection against pathogens [12], but insecticide resistance is a trait for which only recent evidence has indicated a role of symbionts. The bean bug *Riptortus pedestris* and related stinkbug species acquire bacteria of the genus Burkholderia from the soil which then replace the normal Burkholderia midgut symbiont, conferring resistance to the insecticide fenitrothion [13]. Other studies focusing on Bt have also suggested a link between microbial symbionts and the level of host susceptibility, though the current level of knowledge is too basic to indicate a common or more generally applicable mechanism. Susceptibility to Bt and its toxins amongst lepidopteran species was investigated using antibiotic treatment and later oral administration of an indigenous gut bacterium and showed that the presence of a gut microbiota strongly affected susceptibility but in an inconsistent manner across species [14]. Other work on *Spodoptera exigua,* another lepidopteran, indicated that Bt resistance was associated with a higher microbiota load [15].

Many studies have focused on DBM, covering the mechanisms of pesticide resistance [3], [16], [17], developmental biology [18], herbivory mechanism [19], and pest management strategies [20], [21]. Little is known, however, about the roles of DBM symbionts. This is an important gap in knowledge given the importance of such microorganisms to other insects. In addition, the reliance until recently on culture-based methods for the study of microbial symbionts has likely provided an incomplete view of the phylogenetic diversity of bacteria within the DBM midgut [22]. To date, no study has focused on the potential role of midgut symbiotic microorganisms of DBM larvae in insecticidal resistance. In recent years, the culture-independent method of PCR to amplify 16S rRNA has become a powerful tool for investigating the structure of gut bacterial diversity [23]. The development of new sequencing technology facilitates the utilization of the hyper variable regions of 16S rRNA, such as the V3 or V6 regions, which can provide valuable phylogenetic information on bacteria sampled from insects [24], [25], [26], [27].

In the current study, the composition of bacterial communities of the 3^rd^-instar DBM midguts in susceptible, chlorpyrifos- and fipronil-resistant lines were examined using Illumina high-throughput sequencing of the 16S rRNA sampled from an insect generation that was not exposed to insecticide. A complementary experiment used the same approach to study the microbiota of DBM lines treated with insecticides in the study generation. Major differences in both studies in the composition of the midgut bacterial communities are discussed in relation to possible functions of microbial taxa about their potential role in DBM insecticide resistance.

## Materials and Methods

### Comparison of the DBM Midgut Microbes between Susceptible and Resistant Lines Using Individuals from a Generation not Exposed to Insecticides

A DBM strain (Fuzhou-S) was collected in July 2004 from a vegetable field in Fuzhou (26.08°N, 119.28°E), Fujian province, south-eastern China. All necessary permits were obtained prior this study from the Institute of Plant Protection of the Fujian Academy of Agricultural Sciences. Two insecticide-resistant lines were selected from the susceptible strain (SS) by treatment in each generation with either chlorpyrifos or fipronil. Chlorpyrifos is an organophosphate insecticide that acts on the nervous system of insects by inhibiting acetylcholinesterase [28]. Fipronil is a broad spectrum insecticide that disrupts the insect central nervous system by blocking the passage of chloride ions through the GABA receptor [29]. The median lethal concentrations (LC_50_) of the chlorpyrifos-resistant (CRL) and fipronil-resistant lines (FRL) were 574 fold (51,500.00 mg·L^−1^ vs. 89.79 mg·L^−1^) and 72 fold (16.85 mg·L^−1^ vs. 0.23 mg·L^−1^) higher than in the SS, respectively. The three DBM lines were reared on radish seedlings at 25±2°C, 70–80% RH and a 16 h light/8 h dark photoperiod. Insects sampled for microbiota studies were from a generation of each line that was not exposed to any insecticide.

### Study of a DBM Generation Exposed to Insecticide

After hatching, larvae of the two insecticide resistant lines were fed on radish seedlings that were treated with one of the following dosages: 6.0 g·L^−1^, 8.0 g·L^−1^ chlorpyrifos, or 1.0 mg·L^−1^, 2.0 mg·L^−1^ fipronil at 25±2°C, 70–80% RH and a 16 h light/8 h dark photoperiod. This produced cohorts of DBM, designated CRL6.0, CRL8.0, FRL1.0, and FRL2.0, corresponding to the insecticide type and concentration, for comparison with the CRL and FRL insects.

### Collecting Larval Midgut Contents

To collect the midgut contents, 50 3^rd^-instar larvae were randomly sampled from each insect line, regardless of sex. The larvae were surface-sterilized with 75% ethanol for 90 sec and rinsed with sterilized-deionized water. After dissection, the midgut contents were homogenized with 1 ml sterile deionized water and frozen at −80°C before DNA extraction.

### DNA Extraction and PCR Amplification of the V6 Region of 16S rRNA

Total bacterial DNA from the DBM larval midgut was extracted using the PowerSoil®DNA Isolation Kit (MO BIO laboratories, San Diego, USA) following the manufacturer’s protocol with the following changes. The midgut contents were placed into liquid nitrogen and thawed at 37°C before cell lysis. After adding C1 solution (a component of the Kit), the sample was completely homogenized by 20 min of vortexing. Other subsequent steps were performed following the manufacturer’s protocol. The DNA products were run on 1.0% agarose gels.

To amplify 16S rRNA for Illumina deep sequencing, universal primers targeting the V6 region, V6F: 5′- CAACG CGARG AACCT TACC -3′, V6R: 5′- CGACA GCCAT GCASC ACCT -3′, were designed as described previously [30], [31], [32]. The PCR was carried out in a total volume of 20 µL: H_2_O 13.25 µL, 10×PCR ExTaq Buffer 2.0 µL, DNA template (100 ng/µL) 0.5 µL, V6F (10 mmol/L) 1.0 µL, V6R (10 mmol/L) 1.0 µL, dNTP 2.0 µL, ExTaq (5U/µL) 0.25 µL. After initial denaturation at 95°C for 5 min, amplification was performed using 30 cycles of 30 sec at 95°C, 20 sec at 58°C, 6 sec at 72°C, followed by a final extension at 72°C for 7 min. Amplification products were then run on 1.0% agarose gels and purified, and the products were sent to the Beijing Genomics Institute (BGI) in Shenzhen to construct the V6 library for sequencing.

### Deep Sequencing of 16S rRNA V6 Region and Data Analysis

The PCR products were purified and end-repaired, A-tailed, PE-adapter ligated and then sequenced using the 100 bp paired end strategy on the Illumina HiSeq 2000. Clean data were generated after trimming and removing reads with low quality scores, and then, PE reads were overlapped to full V6 tags with a minimum overlap length of 30 bp. Tags with lengths less than 55 bp were removed for further analysis. The redundant tags were deleted by Mothur v.1.11.0 [33], and the unique tags (non-redundant tags) were obtained. The unique tags were aligned against the 16S rRNA V6 database [27] using the BLASTN algorithm with the e-value<1e-5. Taxonomic classification was performed using a two-thirds (66%) majority rule [34]. As a result of the two-thirds majority rule, and the limitation of 16S rRNA V6 database taxonomic information, most tags (>85%) of all the samples were classified into different orders and higher taxa, but a few tags were assigned to families and lower classifications (**Figure S1**). To sufficiently utilize tags, subsequent analysis on the bacterial community was conducted at the order level. After obtaining the unique tags, they were clustered at 97% sequence similarity according to Mothur v.1.11.0 to acquire the target Operational Taxonomic Units (OTUs). Estimates of the diversity of the DBM midgut microbiota, the rarefaction and alpha diversity indices, including the species diversity index of Chao1 [35] and Simpson [36] index, were calculated according to Mothur v.1.11.0.

The overlapped full V6 tags generated from PE reads for each line described herein have been deposited at the SRA (Sequence Read Archive) database under accession numbers: SRR689271, SRR689331, SRR689340, SRR689353, SRR689372, SRR689374, and SRR689397 for SS, CRL, FRL, CRL6.0, CRL8.0, FRL1.0 and FRL2.0 respectively.

### Phylogenetic Analysis

One representative sequence was chosen for each of the OTUs with the abundance higher than 0.05% in the samples of SS, CRL and FRL according to Mothur v.1.11.0. A homologue search was performed using the Basic Local Alignment Search Tool (BLAST) program and the 16S rRNA V6 database [27], and then the homologue sequences were downloaded. Using the MEGA5.0 program, the 60 bp representative sequences were aligned by the ClustalW with default settings, and a phylogenetic analysis was performed based on the neighbor-joining method [37]. The evolutionary distance was measured by the maximum composite likelihood method, and 100 bootstrap replications were used to construct the phylogenetic tree.

### Quantitative PCR

Quantiative PCR (qPCR) was performed to validate the results of Illumina sequencing. qPCR was performed on the SS, FRL, CRL, FRL2.0, and CRL8.0 DBM lines by targeting the phylum specific (Gamma-Proteobacteria and Firmicutes) 16S-rRNA genes with SYBR-green I. The entire bacterial community was quantified in order to calculate the relative abundance of the representative bacteria. The primers for quantifying all the bacteria and the dominant phyla are described in **Table S1**. A 25 µL PCR contained 12.5 µL SYBR® Premix Ex Taq II (Takara Biotechnology (Dalian) Co., Ltd. (Takara Dalian)), 8.5 µL H_2_O, 2 µL genomic DNA template from the representative DBM lines’ midgut symbiotic microorganism (10 ng/µL final concentration), and 1 µL of each primer (10 mmol/L). The qPCR was performed by triplicate in BIO-RAD C1000 Touch™ Thermal Cycler with the procedure as follows: initial denaturation at 95°C for 3 min, followed by 35 cycles of 10 sec at 95°C, 30 sec at 58°C. The relative abundance of the targeted bacteria was calculated by the method of 2^−ΔΔCT^ [38].

## Results

### General DBM Larval Midgut Microbial Diversity

A total of 4.40 Gb of raw data from seven samples was generated. After removing the adaptors, low-quality sequences and overlapping PE reads, 436,562 tags with 28.96 Mb clean data were produced and assigned to bacteria domain. As a result, 1,019 unique tags per sample were produced with an average of 208 operational taxonomic units (OTUs) and 97% identity cutoffs for each sample. A total of 342 OTUs were found but only 4 had a frequency greater that 1% and 21 at a frequency of 0.05%. Although there were some differences in the number of DBM midgut microbiota OTUs among the seven samples (**Table S2**), the quantitative relationship between the number (frequency) of OTUs and the relative OTUs abundance exhibited similar exponential distributions with the majority being rare OTUs (**Figure S2**).

To assess the sequencing depth and the species richness, a rarefaction curve was constructed for each insecticide treatment. The curves exhibited no plateau, although the number of OTUs increased with the number of tags in the DBM 3^rd^-instar midgut microbiota (**Figure S3**). Alpha diversity measurement for the seven samples (**Table S3**) suggested variations in species richness (Chao1) and species evenness (Simpson index) among different samples. Higher species richness of a given sample (e.g., CRL8.0) might present a lower species diversity resulting from a lower evenness of the sample.

### Microbial Diversity in Susceptible DBM

**Figure 1:**
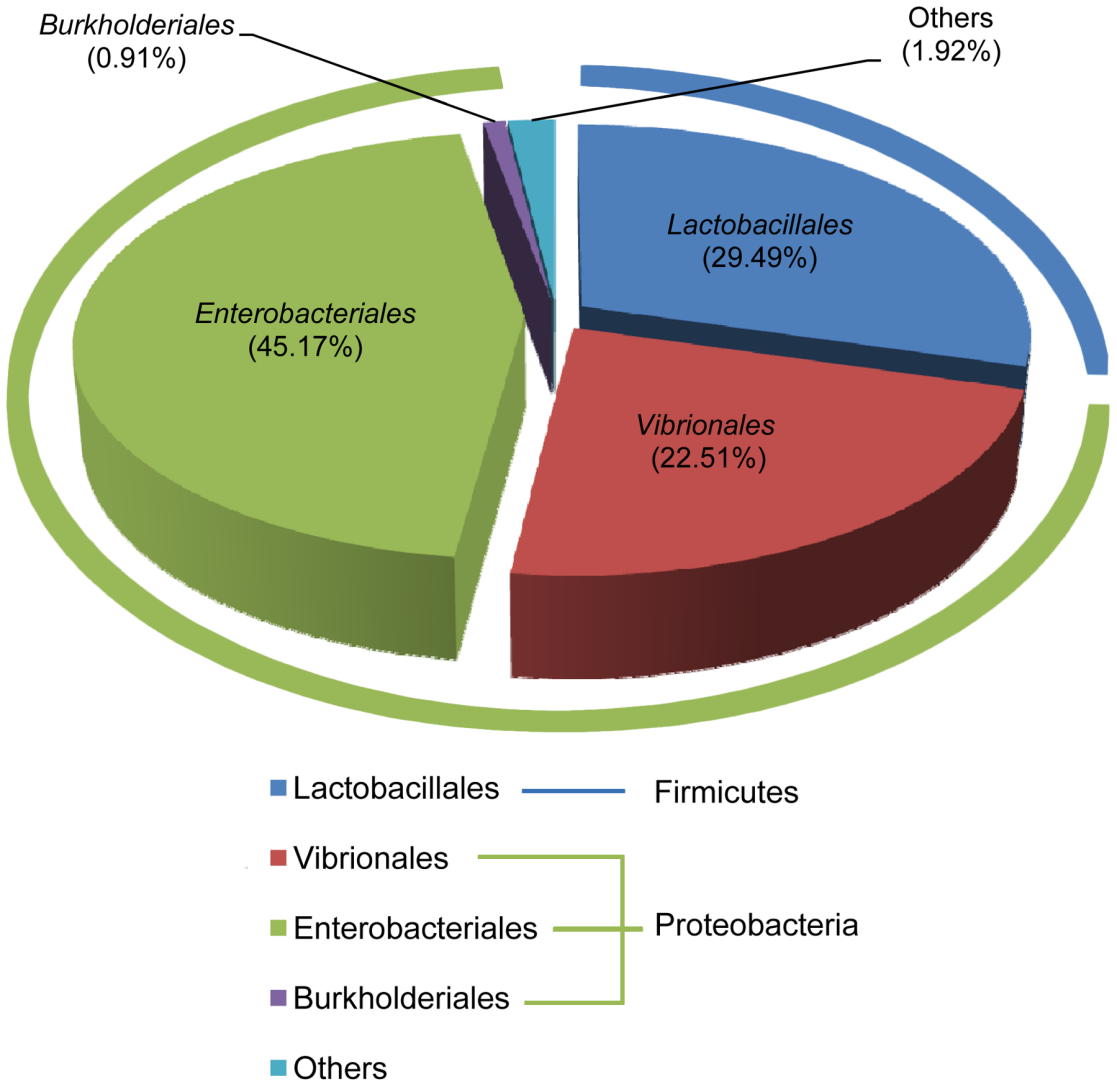
The order-based proportional composition of microbiota in the DBM larval midgut.

In the SS DBM, 150 OTUs were identified. The proportional composition of microbiota in the susceptible larval midgut of SS DBM showed that the midgut was dominated by three orders of bacteria: Enterobacteriales (Phylum Proteobacteria) with a proportion of 45.17% in the total tags, Vibrionales (Phylum Proteobacteria) with 22.51%, and Lactobacillales (Phylum Firmicutes) with 29.49%. These three taxa comprised 97.17% of the DBM larval midgut bacteria (**Figure 1**). It is noteworthy that in each order there were only a few OTUs with high frequencies. For example, in Enterobacteriales, only 4 OTUs had a frequency greater than 0.05%, 1 for Vibrionales, and 2 for Lactobacillales.

The Proteobacteria within the DBM larval midgut included alpha-Proteobacteria (1.67% of the total midgut bacteria), which was subdivided into eight families (Caulobacteraceae, Bradyrhizobiaceae, Hyphomicrobiaceae, Methylobacteriaceae, Rhizobiaceae, Acetobacteraceae, Rhodospirillaceae and Sphingomonadaceae); beta-Proteobacteria (0.91%), consisting of four families (Alcaligenaceae, Burkholderiaceae, Comamonadaceae and Oxalobacteraceae); delta-Proteobacteria (0.01%), including only two families (Bdellovibrionaceae and Polyangiaceae) and Gamma-Proteobacteria (67.76%) subdivided into six families (Aeromonadaceae, Enterobacteriaceae, Moraxellaceae, Pseudomonadaceae, Vibrionaceae and Xanthomonadaceae), in which Enterobacteriaceae and Vibrionaceae dominated the class. The second most abundant phylum in the DBM midgut was Firmicutes, consisting of three orders: Lactobacillales (the most abundant), Bacillales and Clostridiales (**Figure 1** **)**.

### Comparison of the DBM Midgut Microbes between Susceptible and Resistant Lines Using Individuals from a Generation not Exposed to Insecticides

**Figure 2:**
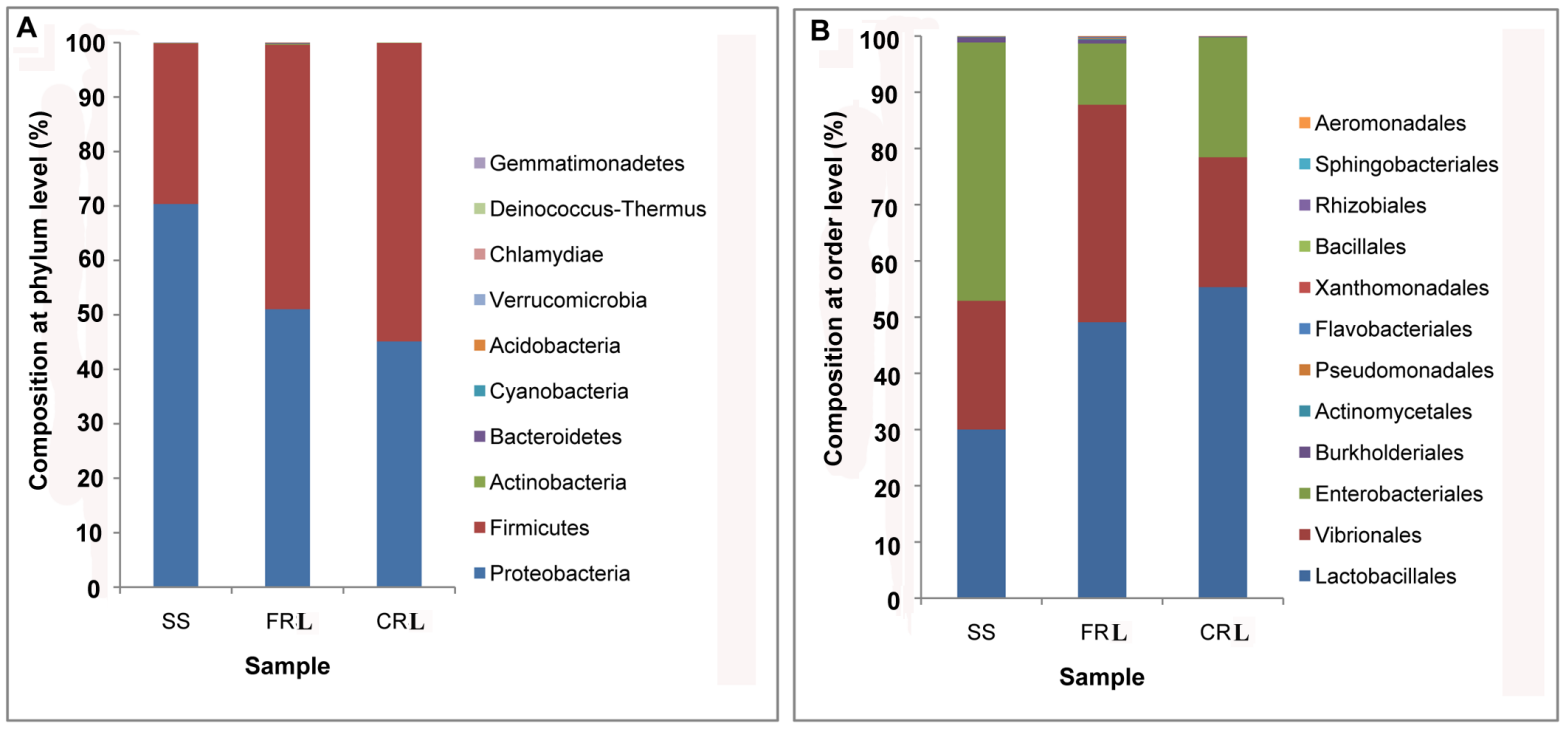
Proportional composition of microbes in the DBM larval midgut for the susceptible strain (SS), chlorpyrifos-resistant line (CRL), and fipronil-resistant line (FRL) not exposed to insecticides. (A) Composition at the phylum level, and (B) composition at the order level.

Two phyla of bacteria (Firmicutes and Proteobacteria) that were dominant in the midgut of the SS strain were also present in the midgut of both insecticide-resistant lines (CRL and FRL), but their proportions were markedly different **(** **Figure 2A** **)**. The frequencies of Firmicutes increased (from 29.52% in SS to 54.79% in CRL and 48.56% in FRL) while frequencies of Proteobacteria decreased (from 70.4% in SS to 45.13% in CRL and 51.05% in FRL). These differences were also marked in the qPCR validation experiment with the insecticide-resistant lines exhibiting a higher proportion of bacteria from the phylum of Firmicutes and a lower proportion of Proteobacteria than the SS strain (**Figure S4**).

**Figure 3:**
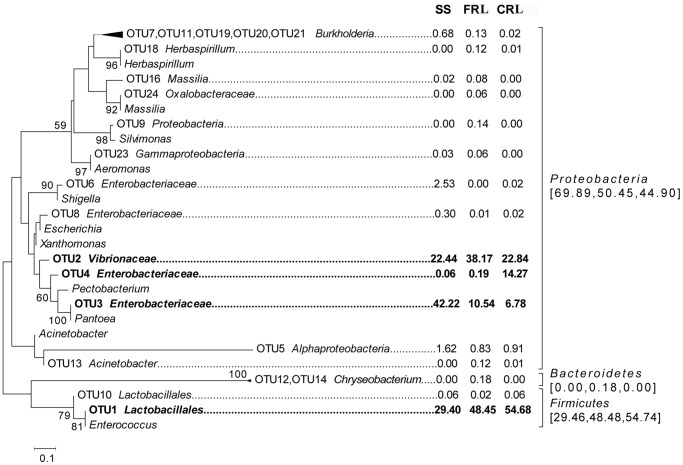
Phylogenetic analysis based on the 16S rRNA V6 region sequences from samples of the susceptible strain (SS), chlorpyrifos-resistant lines (CRL) and fipronil-resistant lines (FRL). The bootstrap value (100 replicates) associated with the taxa clustered in the tree is shown next to the branches. The scaled bar represents 0.1% estimated phylogenetic divergence. The numbers below SS, FRL and CRL are the percent abundances corresponding to the OTUs on the tree, and the numbers in the square brackets are the percentage abundances corresponding to the phylum in SS, FRL and CRL.

At the order level, Enterobacteriales and Vibrionales in Proteobacteria, and Lactobacillales in Firmicutes remained dominant (**Figure 2B**). Phylogenetic trees of SS, FRL, and CRL showed that Enterobacteriales, Vibrionales, and Lactobacillales were the dominant orders (**Figure 3**). Of the total microbes in SS, 29.49% were identified as Lactobacillales, 45.17% as Enterobacteriales, and 22.51% as Vibrionales, while in CRL and FRL, 54.76% and 48.51% were identified as Lactobacillales, 21.13% and 10.77% as Enterobacteriales, and 22.89% and 38.21% as Vibrionales, respectively.

Some of the least abundant orders increased in frequency from the SS to the insecticide resistant lines. For example, the frequency of Pseudomonadales was 0.025% of the total microbes in SS yet 0.427% in FRL lines and 0.772% in the CRL lines. Similarly, Xanthomonadales increased from 0.016% in SS to 0.104% in FRL lines and 0.237% in CRL lines (**Figure S5**).

### Study of a DBM Generation Exposed to Insecticide

**Figure 4:**
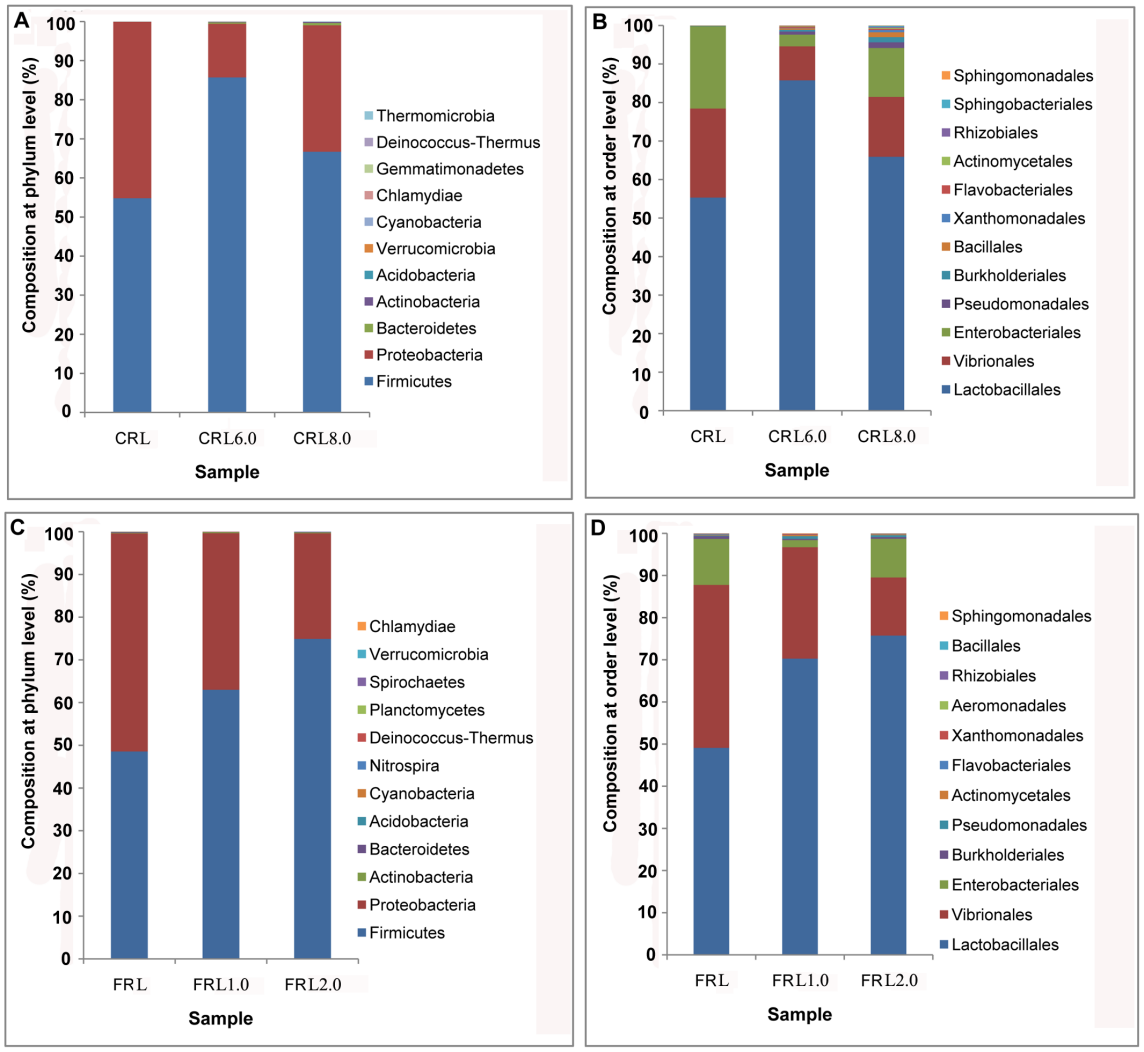
Proportional composition of microbes in the DBM larval midgut of individuals from the chlorpyrifos-resistant (CRL) and fipronil-resistant (FRL) communities reared under insecticide-stressed conditions. Compositions of (A) CRL at the phylum level, (B) CRL at the order level, (C) FRL at the phylum level, and (D) FRL at the order level.

Exposure to insecticides led to a change in the frequencies of some of the major phyla in the DBM midgut microbiota. Firmicutes and Proteobacteria remained dominant in all insect cohorts. The exposure of DBM to different dosages of insecticides led to a lower proportion of Proteobacteria and a higher proportion of Firmicutes in CRL6.0, CRL8.0, FRL1.0 and FRL2.0 than in CRL and FRL **(** **Figure 4** **)**. For example, the proportion of the phylum Firmicutes in FRL1.0 and FRL2.0 was 14.45% and 26.36% higher, respectively, than FRL, and 30.89% and 11.89% higher in CRL6.0 and CRL8.0, respectively, than CRL. The proportion of Proteobacteria in CRL6.0 and CRL8.0 was 31.41% and 12.82% lower, respectively, than CRL, and 14.41% and 26.33% lower in FRL1.0 and FRL2.0, respectively, than FRL (**Figures 4A and 4C**). At the order level (**Figures 4B and 4D**), the proportion of Lactobacillales was 30.93% and 10.58% higher in CRL6.0 and CRL8.0, respectively, than CRL, and 14.48% and 26.35% higher in FRL1.0 and FRL2.0, than FRL. These results showed similar trends in the proportions of microbes in the midgut of DBM larvae coming from IRLs and SS, i.e. the abundance of Firmicutes (phylum level) and Lactobacillales (order level) were higher in the IRLs than SS. The qPCR experiment also showed that the relative abundance of Firmicutes increased in the resistant lines (CRL8.0, FRL2.0) when exposed to insecticides, compared with the resistant lines (CRL, FRL) without exposure to insecticides (**Figure S4**).

## Discussion

The results of our study showed that the midgut microbiota of DBM was diverse but dominated by only two microbial phyla (Proteobacteria and Firmicutes), with a dominance of more than 97%. There are very few equivalent studies but that by Osei-Poku*et al*. found similar results with only one taxon dominating (90%) in eight species of mosquitoes [39]. Previous studies of insect midgut microbiology also found that these two phyla (Proteobacteria and Firmicutes) were dominant in the midgut of Lepidoptera, including *Lymantria dispar*, *Helicoverpa armigera*, and *Bombyx mori* [40], [41], [42]. At the order level, the most abundant order in the DBM midgut was Enterobacteriaceae. It has been suggested that the capacity of Enterobacteriaceae to degrade carbohydrates may be useful for digestion by the host insect [43] and this function should be tested for DBM.

The differences in midgut bacterial composition between susceptible and insecticide-resistant lines that had a common origin prior to insecticide exposure treatment were large. While these could be a consequence of the insecticide treatments causing differential toxicity to different bacterial taxa, it is important to consider the possibility that the altered biota of the insecticide resistant lines and cohorts exposed to insecticides are an indication of a role of certain bacteria in conferring resistance. The insecticide resistant lines exhibited a higher proportion of bacteria from the order of Lactobacillales (phylum Firmicutes) and a commensurate reduction in the proportion of Enterobacteriaceae (phylum Proteobacteria) compared to the susceptible strain (SS). These differences could point to adaptive responses in biota to the new chemical environment of the midgut. In the case of a role in insecticide resistance, Kikuchi *et al*. demonstrated symbiont-mediated insecticide resistance in stinkbugs [13]. *Riptortus pedestris* acquires symbiotic *Burkholderia* from the soil and laboratory studies have shown that the process to be very efficient with an infective dose of just 80 cells [44]. Fenitrothion application to the soil selects fenitrothion-degrading strains of *Burkholderia* and immature *R. pedestris* in treated plots show high levels of acquisition of some such strains [13]. Remarkably, establishment in the stinkbug gut of the fenitrothion-degrading *Burkholderia* strains conferred high levels of resistance to percutaneous as well as oral treatment with the insecticide compared with insects infected by non-fenitorthion-degrading strains of the same bacterium. That finding illustrates the importance of strain-level differences in microbionts, so the differences in higher level taxa (order and phylum) in the present study demand further work to identify the causal lower-level taxa as well as to confirm the phenomenon.

Given the significance of DBM as a global pest, and the fact that insecticide resistance makes it difficult to manage, the possibility that microbial symbiont-mediated resistance, especially by Lactobacillales, applies in the case of this insect needs to be further investigated. We also found that some of the less abundant microbiotia identified by Illumina sequencing varied markedly between susceptible and insecticide resistant lines. The possibility that they play a role in insecticide resistance in DBM, rather than their relative abundance being a consequence of the insecticide, remains to be tested. Further studies need to be completed to determine their roles in the nutrition of the host and potential roles in insecticide resistance.

Currently, there is insufficient evidence to establish whether certain bacterial taxa are be responsible for conferring insecticide resistance in DBM and whether such a mechanism acts in concert with other mechanisms such as changes in insect physiology that induce or facilitate the microbiota community to change. Under either scenario, the present results open an avenue for the molecular study of insect midgut microbiota and its relationship with the important phenomenon of insecticide resistance. Potentially, an understanding of the relevant mechanisms and symbiont taxa could lead to more active forms of toxicant and novel approaches for insecticide resistance management in pests such as DBM.

## Supporting Information

Figure S1 **Taxonomic distribution of assigned V6 tag sequences.** Bar represents the number of tags for different taxa in each of the samples.
(TIF)

Figure S2 **Numerical distribution against abundance of the OTUs of microbiota in the larval midgut of DBM, calculated by Mothur v.1.11.0.** The abscissa represents the OTU abundance, and the ordinate represents the number of the OTUs corresponding to the abundance. The number of the OTUs is numerically accumulated when the OTU abundance is greater than 15, and presented in the abundance of 15.
(TIF)

Figure S3 **The rarefaction curve describing the number of the OTUs observed against the number of the tags sampled in the larval midgut microbiota of DBM.**
(TIF)

Figure S4 **qPCR analysis of the relative abundance of microbes at phylum level in the DBM larval midgut of individuals from the susceptible strain (SS), chlorpyrifos-resistant (CRL), fipronil-resistant (FRL) lines, and CRL and FRL reared under insecticide-stressed conditions.**
(TIF)

Figure S5 **The frequencies of Pseudomonadales and Xanthomonadales variation between susceptible DBM strain and insecticide resistant lines.**
(TIF)

Table S1 **The phylum-specific 16S rRNA qPCR primers.**
(DOCX)

Table S2 **Number of the OTUs of microbiota in the larval midgut of DBM, calculated by Mothur v.1.11.0.**
(DOCX)

Table S3 **Alpha diversity of microbiota in the larval midgut of DBM.**
(DOCX)
