# Supplementary figures and images for: DNA Sequencing Reveals the Midgut Microbiota of Diamondback Moth, Plutella xylostella (L.) and a Possible Relationship with Insecticide Resistance
Source: PLoS One. 2013 Jul 19;8(7):e68852. doi: 10.1371/journal.pone.0068852 (PMC3716819; doi:10.1371/journal.pone.0068852)

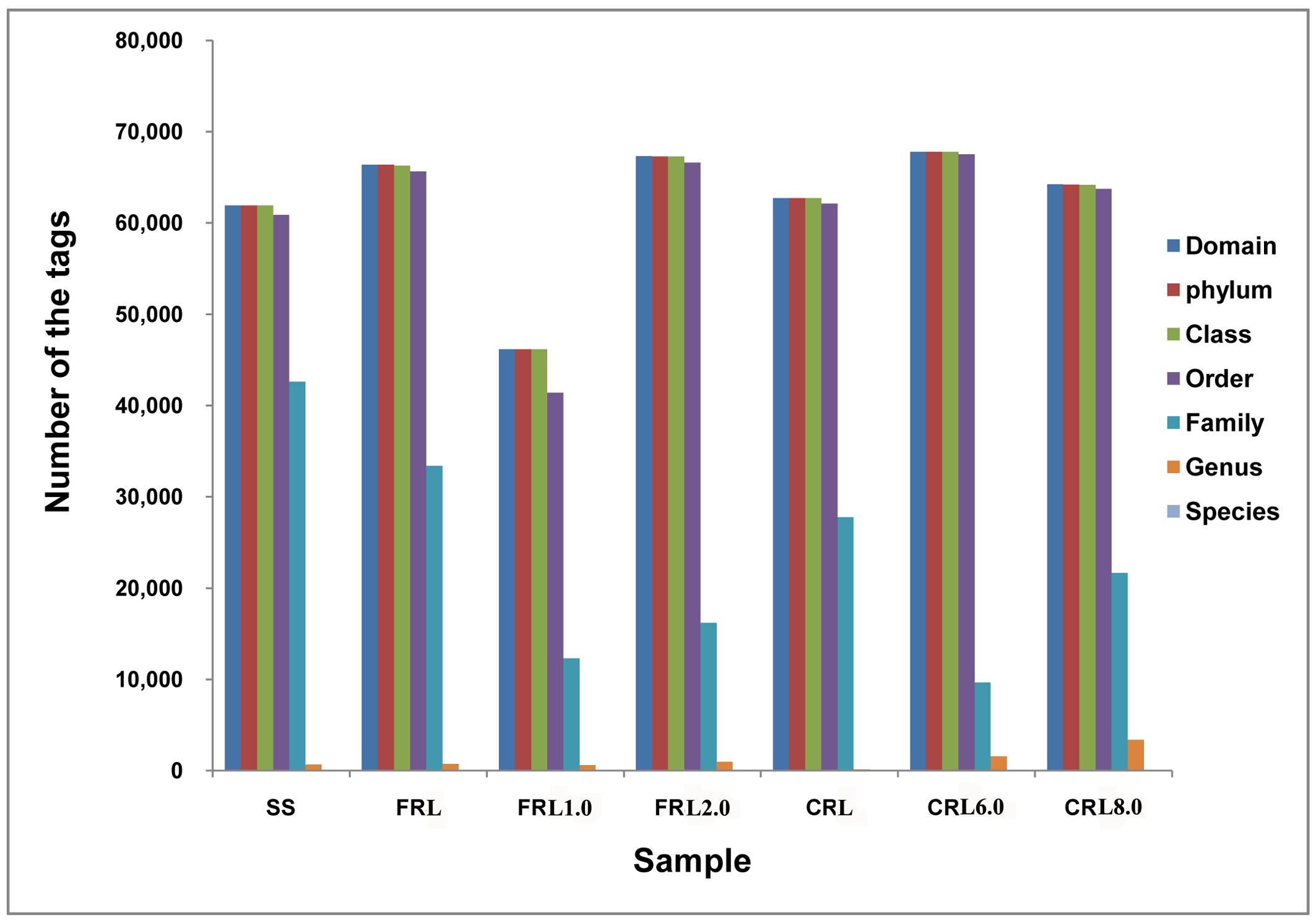

Supplement: Figure S1 — Taxonomic distribution of assigned V6 tag sequences. Bar represents the number of tags for different taxa in each of the samples. (TIF) [file pone.0068852.s001.tif]

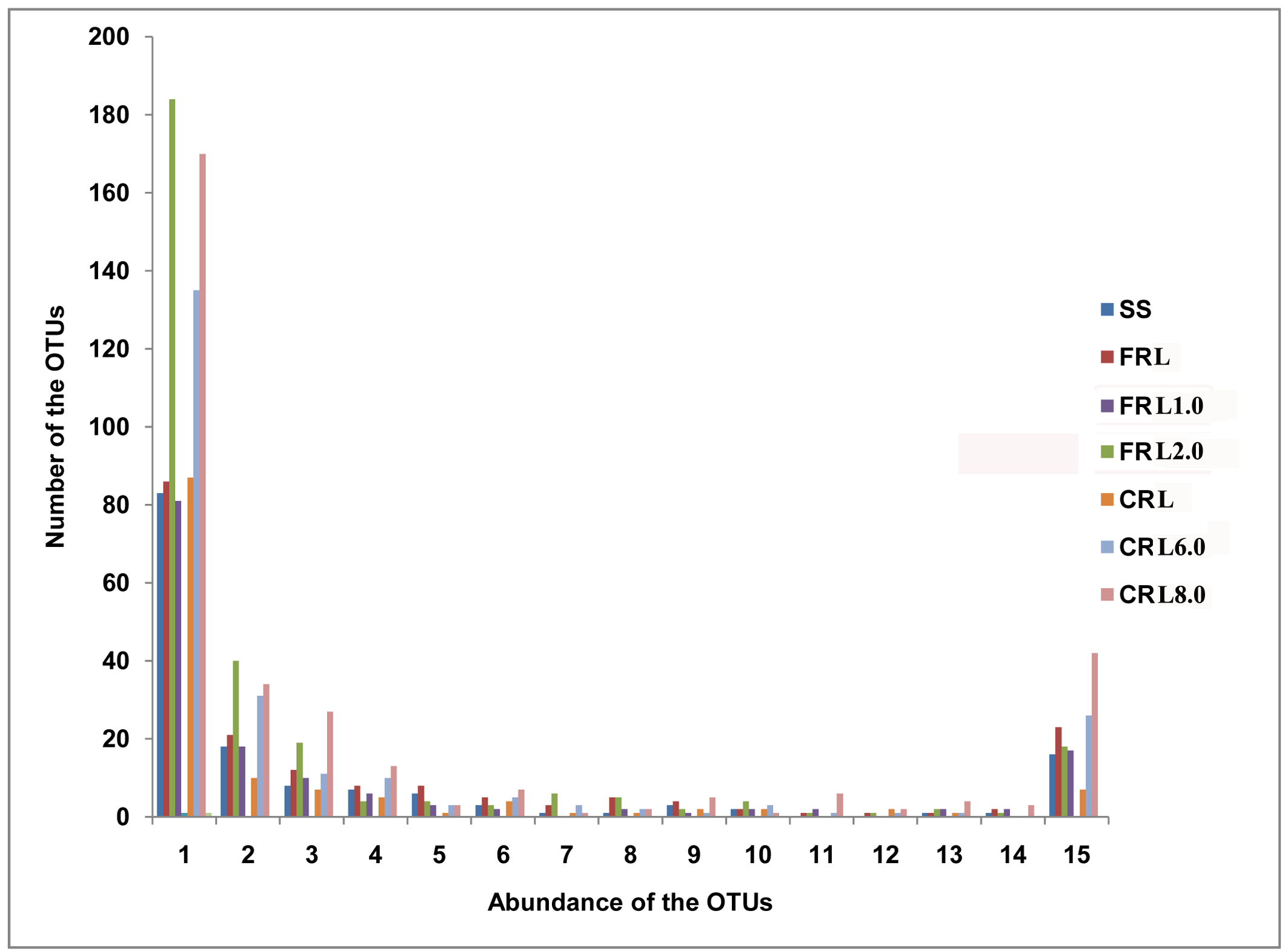

Supplement: Figure S2 — Numerical distribution against abundance of the OTUs of microbiota in the larval midgut of DBM, calculated by Mothur v.1.11.0. The abscissa represents the OTU abundance, and the ordinate represents the number of the OTUs corresponding to the abundance. The number of the OTUs is numerically accumulated when the OTU abundance is greater than 15, and presented in the abundance of 15. (TIF) [file pone.0068852.s002.tif]

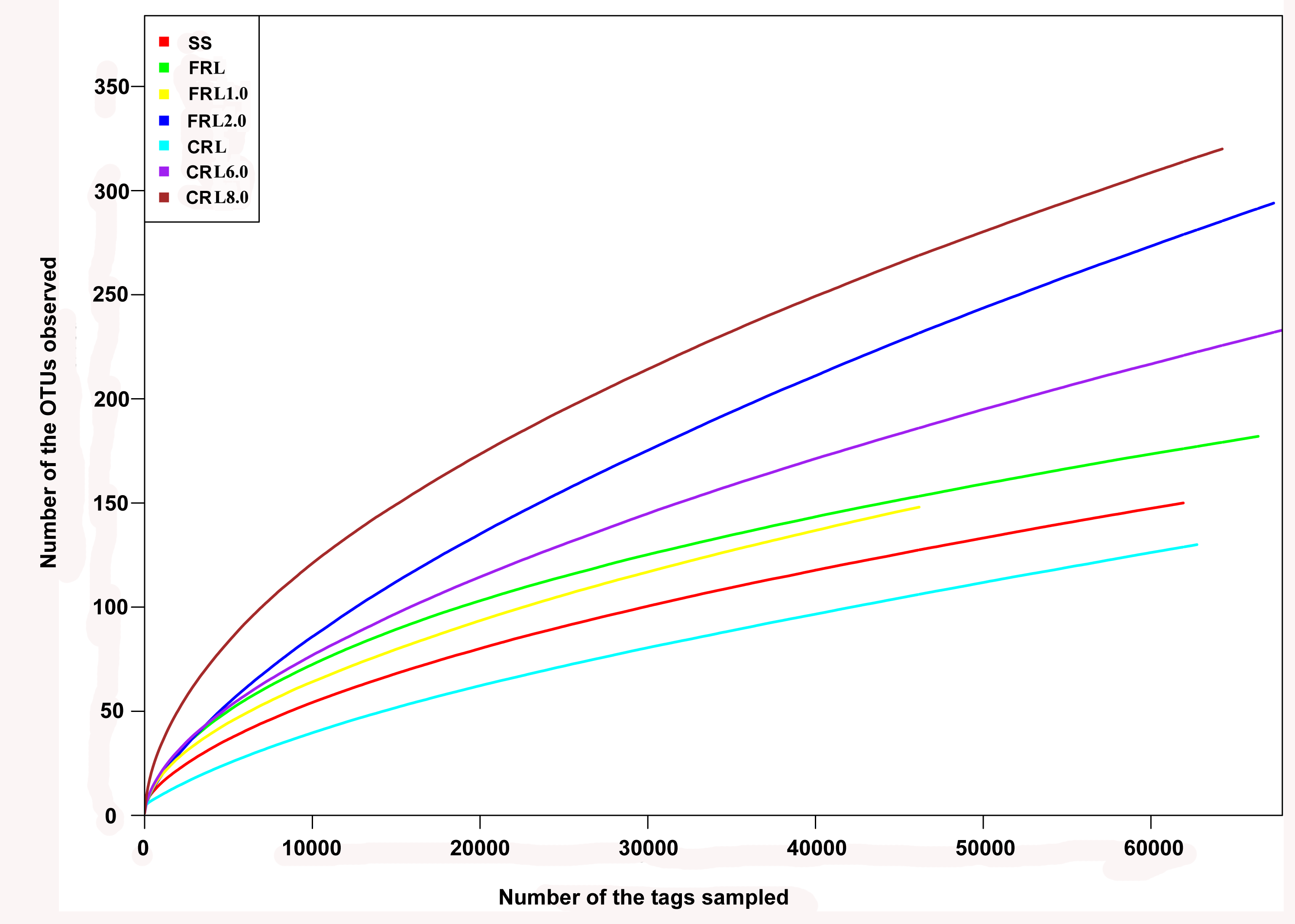

Supplement: Figure S3 — The rarefaction curve describing the number of the OTUs observed against the number of the tags sampled in the larval midgut microbiota of DBM. (TIF) [file pone.0068852.s003.tif]

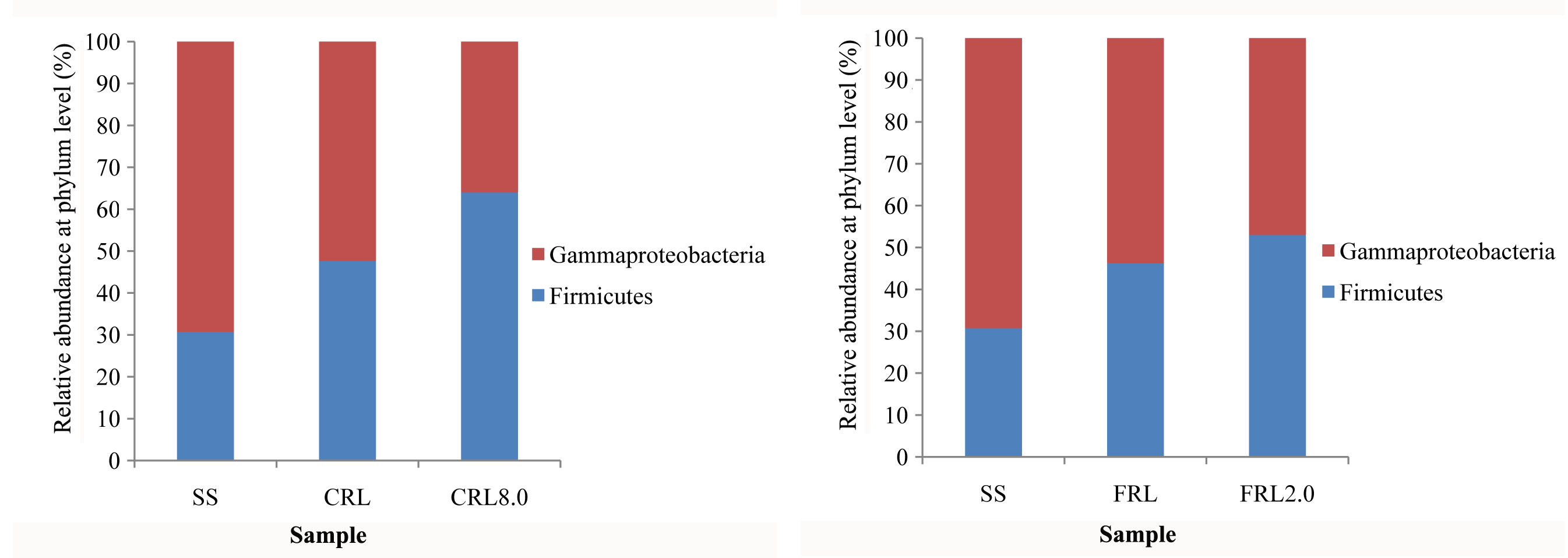

Supplement: Figure S4 — qPCR analysis of the relative abundance of microbes at phylum level in the DBM larval midgut of individuals from the susceptible strain (SS), chlorpyrifos-resistant (CRL), fipronil-resistant (FRL) lines, and CRL and FRL reared under insecticide-stressed conditions. (TIF) [file pone.0068852.s004.tif]

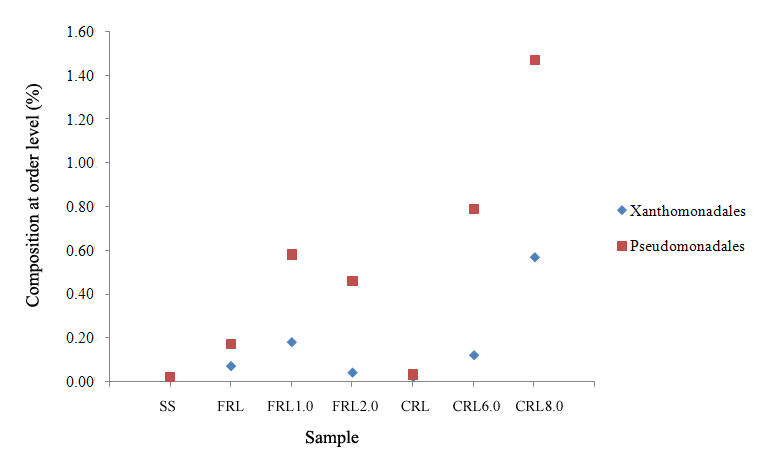

Supplement: Figure S5 — The frequencies of Pseudomonadales and Xanthomonadales variation between susceptible DBM strain and insecticide resistant lines. (TIF) [file pone.0068852.s005.tif]
