# Supplementary material for: DNA Sequencing Reveals the Midgut Microbiota of Diamondback Moth, Plutella xylostella (L.) and a Possible Relationship with Insecticide Resistance
Source: PLoS One. 2013 Jul 19;8(7):e68852. doi: 10.1371/journal.pone.0068852 (PMC3716819; doi:10.1371/journal.pone.0068852)
Supplement: Table S1 — The phylum-specific 16S rRNA qPCR primers. (DOCX) [file pone.0068852.s006.docx]

**Table S1 The phylum-specific 16S rRNA qPCR primers**

| Primers | Taxon | Forward | Reverse | References |
| --- | --- | --- | --- | --- |
| Eub  Fim  GPr | All bacteria  Firmicutes  Gamma-Proteobacteria | 5'- CGGCAACGAGCGCAACCC -3'  5'- GGAGYATGTGGTTTAATTCGAAGCA -3'  5'- TCG TCA GCT CGT GTY GTGA -3' | 5'- CCATTGTAGCACGTGTGTAGCC -3'  5'- AGCTGACGACAACCATGCAC -3'  5'- CGT AAG GGC CAT GATG -3′ | [[1](#_ENREF_1)]  [[2](#_ENREF_2)]  [[3](#_ENREF_3)] |

**References**

1. Denman SE, McSweeney CS (2006) Development of a real-time PCR assay for monitoring anaerobic fungal and cellulolytic bacterial populations within the rumen. *FEMS* *Microbiol Ecol* 58: 572-582.

2. Guo X, Xia X, Tang R, Zhou J, Zhao H, et al. (2008) Development of a real-time PCR method for Firmicutes and Bacteroidetes in faeces and its application to quantify intestinal population of obese and lean pigs. *Lett Appl Microbiol* 47: 367-373.

3. Bacchetti De Gregoris T, Aldred N, Clare AS, Burgess JG (2011) Improvement of phylum- and class-specific primers for real-time PCR quantification of bacterial taxa. *J Microbiol Methods* 86: 351-356.
