# Supplementary material for: DNA Sequencing Reveals the Midgut Microbiota of Diamondback Moth, Plutella xylostella (L.) and a Possible Relationship with Insecticide Resistance
Source: PLoS One. 2013 Jul 19;8(7):e68852. doi: 10.1371/journal.pone.0068852 (PMC3716819; doi:10.1371/journal.pone.0068852)
Supplement: Table S2 — Number of the OTUs of microbiota in the larval midgut of DBM, calculated by Mothur v.1.11.0. (DOCX) [file pone.0068852.s007.docx]

**Table S2 Number of the OTUs of microbiota in the larval midgut of DBM, calculated by Mothur v.1.11.0**

| **Sample** | **Threshold** | **Number of OTUs** |
| --- | --- | --- |
| CRL | 0.03 | 130 |
| CRL6.0 | 0.03 | 233 |
| CRL8.0 | 0.03 | 320 |
| SS | 0.03 | 150 |
| FRL | 0.03 | 182 |
| FRL1.0 | 0.03 | 148 |
| FRL2.0 | 0.03 | 294 |
