# Supplementary material for: DNA Sequencing Reveals the Midgut Microbiota of Diamondback Moth, Plutella xylostella (L.) and a Possible Relationship with Insecticide Resistance
Source: PLoS One. 2013 Jul 19;8(7):e68852. doi: 10.1371/journal.pone.0068852 (PMC3716819; doi:10.1371/journal.pone.0068852)
Supplement: Table S3 — Alpha diversity of microbiota in the larval midgut of DBM. (DOCX) [file pone.0068852.s008.docx]

**Table S3 Alpha diversity of microbiota in the larval midgut of DBM**

| **Sample** | **Alpha diversity** | | |
| --- | --- | --- | --- |
|  | **chao1** | **Simpson** |  |
| CRL | 470 | 0.376 |  |
| CRL6.0 | 516 | 0.735 |  |
| CRL8.0 | 730 | 0.465 |  |
| SS | 329 | 0.316 |  |
| FRL | 348 | 0.392 |  |
| FRL1.0 | 319 | 0.463 |  |
| FRL2.0 | 705 | 0.585 |  |
